# Developing a Novel Fermented Milk with Anti-Aging and Anti-Oxidative Properties Using *Lactobacillus kefiranofaciens* HL1 and *Lactococcus lactis* APL015

**DOI:** 10.3390/nu17152447

**Published:** 2025-07-27

**Authors:** Sheng-Yao Wang, Wei-Chen Yen, Yen-Po Chen, Jia-Shian Shiu, Ming-Ju Chen

**Affiliations:** 1 Department of Animal Science and Technology, National Taiwan University, Taipei 10617, Taiwan; yaoyao@ntu.edu.tw (S.-Y.W.); willy840730@gmail.com (W.-C.Y.); 2 Department of Animal Science, National Chung Hsing University, Taichung 40227, Taiwan; 3 The iEGG and Animal Biotechnology Research Center, National Chung Hsing University, Taichung 40227, Taiwan; 4 Southern Region Branch, Livestock Research Institute, Ministry of Agriculture, Pingtung 946, Taiwan; mucleshank12@gmail.com

**Keywords:** probiotics, fermented milk, *Lactobacillus kefiranofaciens*, *Lactococcus lactis*, oxidative stress, inflammation, gut–brain axis, D-galactose aging

## Abstract

**Background/Objectives:** *Lactobacillus kefiranofaciens* HL1, isolated from kefir, exhibits antioxidant and anti-aging activities, defined here as improved cognitive function and reductions in oxidative stress and inflammatory markers. However, its poor milk viability limits application. This study developed a novel fermented milk by co-culturing HL1 with *Lactococcus lactis* subsp. *cremoris* APL015 (APL15) to enhance fermentation and health benefits. **Methods:** HL1 and APL15 were co-cultured to produce fermented milk (FM), and fermentation performance, microbial viability, texture, and syneresis were evaluated. A D-galactose-induced aging BALB/c mouse model was used to assess cognitive function, oxidative stress, inflammation, antioxidant enzyme activity, and gut microbiota after 8 weeks of oral administration. **Results:** FM reached pH 4.6 within 16 h, with high viable counts (~10^9^ CFU/mL) for both strains. HL1 viability and texture were maintained, with smooth consistency and low syneresis. In vivo, FM improved cognitive behavior (Y-maze, Morris water maze), reduced oxidative damage (MDA), lowered IL-1β and TNF-α, and enhanced brain SOD levels. FM-fed mice exhibited increased short-chain fatty acid producers, higher cecal butyrate, and reduced *Clostridium perfringens*. **Conclusions:** The co-cultured fermented milk effectively delivers HL1 and provides antioxidant, anti-inflammatory, and anti-aging effects in vivo, likely via gut–brain axis modulation. It shows promise as a functional food for healthy aging.

## 1. Introduction

The global population is rapidly aging, bringing increased prevalence of chronic diseases and cognitive decline. Aging is strongly associated with cumulative oxidative stress and chronic inflammation, which contribute to tissue damage and neurodegeneration [1,2]. Targeting oxidative stress with dietary anti-oxidants or probiotics has emerged as a promising strategy to mitigate aging-related health issues [3,4]. In particular, the gut–brain axis has gained attention for its role in regulating aging processes: imbalances in gut microbiota (dysbiosis) can exacerbate systemic inflammation and even influence brain aging and cognition via immune and neural pathways [5,6]. Thus, interventions that modulate the gut microbiome may improve not only gut health but also brain function in the elderly [7].

Fermented dairy products are ideal vehicles for delivering probiotic bacteria with health benefits. Traditional fermented milks such as kefir have long been regarded as health-promoting. Kefir grains harbor unique lactic acid bacteria and yeasts; among these, *Lactobacillus kefiranofaciens* is notable for producing an exopolysaccharide (kefiran) that enhances viscosity and may impart bioactive properties [8]. A strain of this species, *Lb. kefiranofaciens* HL1 was isolated from Taiwanese kefir [9] and found to possess strong anti-oxidant capacity in vitro (DPPH radical scavenging, Fe^2+^ chelation) [10]. Importantly, feeding *Lb. kefiranofaciens* HL1 to D-galactose-induced aging mice improved their resistance to oxidative stress, enhanced learning and memory performance, and beneficially modulated gut microbiota [11]. These findings indicate HL1’s potential as an anti-aging probiotic. However, incorporating HL1 into functional foods is challenging because it does not thrive in milk culture on its own. It has a slow growth rate and struggles to acidify milk, resulting in poor viability during fermentation. This limits the direct use of HL1 in yogurt or fermented milk production.

Co-culture strategies offer a solution for implementing such probiotics in foods. By fermenting milk with a compatible starter culture alongside the probiotic, one can achieve proper fermentation (acid production and texture formation) while maintaining the probiotic’s viability [12,13]. Prior studies have shown that co-culturing certain *Lactobacillus* or *Bifidobacterium* strains with conventional yogurt starters improves the probiotics’ survival and product quality [14,15]. *Lactococcus lactis* subsp. *cremoris* is a mesophilic starter widely used in fermented milks (in Scandinavian fermented milk “viili” and other ropy fermented products) and is known for producing exopolysaccharides that enhance texture and potentially confer health benefits [16,17]. In particular, ropy strains of *Lc. lactis* can increase viscosity and has been reported to stimulate immune cells and even lower serum cholesterol in animal studies [17]. We hypothesized that co-culturing *Lb. kefiranofaciens* HL1 with a robust acid-producing *Lc. lactis* strain could yield a symbiotic fermentation: the *Lc. lactis* would rapidly acidify the milk and form the curd, while allowing HL1 to survive and potentially contribute its bioactive metabolites.

In the commercial probiotic market, the most widely used strains include species of *Lacticaseibacillus* (e.g., *L. rhamnosus* GG), *Lactiplantibacillus* (e.g., *L. plantarum*), and *Bifidobacterium* (e.g., *B. animalis* subsp. *lactis* BB-12), in addition to certain strains of *Lactococcus* and *Streptococcus thermophilus*. These strains are commonly incorporated into fermented dairy products and dietary supplements due to their well-documented gastrointestinal, immunomodulatory, and anti-oxidative benefits [18,19]. For example, *Lacticaseibacillus rhamnosus* GG and *Bifidobacterium animalis* BB-12 are among the most extensively studied probiotics for enhancing gut barrier function and immune responses [18]. Moreover, *Lactiplantibacillus plantarum* CQPC11 has been reported to alleviate cognitive decline and reduce inflammation in aged mice, highlighting its relevance to aging-related health applications [3]. Similarly, the anti-oxidative and gut–brain benefits of *L. casei* Shirota have been demonstrated, particularly in maintaining gut microbiota stability and mitigating stress-induced symptoms [20]. According to recent reports, the global probiotics market exceeded USD 60 billion in 2023, with dairy-based functional foods accounting for approximately 65% of total sales [21]. These data reflect the strong industrial and scientific interest in probiotics with anti-oxidative and anti-aging properties.

However, strains such as *Lb. kefiranofaciens* HL1, despite their promising bioactivities, remain underutilized in commercial applications due to technological limitations in dairy fermentation. In addition, we included the widely used yogurt starters *Streptococcus thermophilus* and *Lactobacillus delbrueckii* subsp. *bulgaricus* as reference cultures in this study. Although traditionally employed for their contributions to acidification and sensory properties, these strains also possess well-established roles in supporting gut microbiota health, making them suitable controls when evaluating potential functional benefits beyond conventional fermentation performance. Our work aims to address the gap in the application of HL1 by exploring co-culture strategies to enhance its technological feasibility.

In this work, we applied a co-culture approach using *Lb. kefiranofaciens* HL1 and *Lc. lactis* subsp. *cremoris* APL015 (aropy EPS-producing strain isolated from fermented milk) to develop a novel fermented milk product. We first evaluated the fermentation performance, viability, and product qualities (texture and sensory attributes) of this co-culture fermented milk, comparing it with fermentation using each strain individually. We then investigated the health effects of the fermented milk in a D-galactose-induced aging mouse model, a well-established model that mimics accelerated aging via chronic oxidative stress and inflammation. We focused on aging-related biomarkers in peripheral tissues and brain, gut microbiota changes, and behavioral outcomes (learning and memory tests) to assess the impact of the fermented milk. We also examined whether delivering HL1 in the fermented milk matrix enhances its anti-aging efficacy compared to HL1 alone. The overall goal was to determine whether this new fermented milk with HL1 and APL015 can serve as a functional food with anti-aging and anti-oxidative properties, and to elucidate the role of the gut–brain axis in mediating its benefits. In this context, the anti-aging effects refer to physiological improvements relevant to cognitive function, oxidative stress, and inflammation.

## 2. Materials and Methods

### 2.1. Bacterial Strains and Culture Conditions

*Lactobacillus kefiranofaciens* HL1, isolated from Taiwanese kefir grains [9], and *Lactococcus lactis* subsp. *cremoris* APL015, isolated from Taiwanese ropy fermented milk [22], was used as the co-culture probiotic and starter strains, respectively. Both strains were identified via 16S rRNA sequencing as previously described [9,22,23], and were maintained in de Man, Rogosa, and Sharpe (MRS) broth with 20% glycerol at −80 °C. Prior to the experiments, the strains were subcultured twice in MRS broth at 30 °C for 24 h under anaerobic (HL1) or aerobic (APL015) conditions [24].

### 2.2. Preparation of Fermented Milk

Reconstituted skim milk (12% *w*/*v*, Foremost, Taipei, Taiwan) was sterilized at 95 °C for 10 min and cooled to 30 °C. Four fermentation setups were prepared: (1) HL1 alone, (2) APL015 alone, (3) HL1 + APL015 co-culture, and (4) yogurt control with commercial starter (*L. delbrueckii* subsp. bulgaricus and *Streptococcus thermophilus*, YC-380, Chr. Hansen, Hørsholm, Denmark). HL1 and/or APL015 were inoculated at 2% (*v*/*v*) each (1:1 ratio for co-culture). Fermentation proceeded statically at 30 °C until pH 4.6 was reached, with the pH and titratable acidity measured periodically.

### 2.3. Microbial Viability and Product Analysis

Viable cell counts were determined by plating serial dilutions on MRS agar (HL1, anaerobic, 30 °C, 72 h) or M17 agar (APL015, aerobic, 30 °C, 48 h). Physical properties including syneresis, viscosity (measured using rotational viscometer (RST-CPS; Brookfield Engineering Laboratories Inc., Middleboro, MA, USA) at 130 s^−1^, 4 °C), and texture (measured using Texture Analyzer (Stable Micro Systems Ltd., Godalming, UK)) were assessed post-fermentation.

### 2.4. Animal Study Design

Male BALB/c mice (8 weeks old, National Laboratory Animal Center, Taipei, Taiwan) were housed under standard conditions (22 ± 2 °C, 50% humidity, 12 h light/dark) with free access to chow and water. D-galactose-induced aging was modeled via the subcutaneous injection of D-galactose (100 mg/kg/day) for 8 weeks. Mice were divided into six groups (n = 10): (1) healthy control (saline), (2) D-gal control, (3) D-gal + HL1, (4) D-gal + APL015, (5) D-gal + APL015 fermented milk, and (6) D-gal + HL1 + APL015 fermented milk. Daily supplementation was given via oral gavage (0.2 mL, ~1 × 10^9^ CFU bacteria per dose). All procedures were approved by the Institutional Animal Care and Use Committee (IACUC, NTU-109-EL-00014).

### 2.5. Behavioral Testing

Cognitive function was assessed using the Y-maze spontaneous alternation [25] and Morris water maze (MWM) tests [26], based on established protocols. Y-maze alternation percentage was calculated over an 8 min trial. For MWM, mice underwent four training trials per day for 4 days (escape latency measured), with a probe trial (platform removed) on day 5 to assess memory retention [27,28]. All assessments were video-recorded, and the researchers were blinded to groups during the analysis.

### 2.6. Biochemical Analysis

Brain, liver, and serum samples were collected post-mortem. Oxidative stress was quantified by measuring malondialdehyde (MDA) using TBARS assay kits and superoxide dismutase (SOD) activity via commercial colorimetric kits (Cayman Chemical Company, Ann Arbor, MI, USA). Inflammatory cytokines (IL-1β, TNF-α) were measured using ELISA (Duoset^®^ ELISA kit, R&D Systems, Inc., Minneapolis, MN, USA). Cecal DNA content was extracted using the QIAamp Stool Kit (QIAGEN, Hilden, Germany). According to the reagent preparation method of a commercial qPCR enzyme kit (KAPA SYBR FAST qPCR Master (2x) kit, Applied Biosystems, Waltham, MA, USA), primers specific to *Lactobacillus* [29], *Enterobacteriaceae* [30], and *Clostridium perfringens* [31] (Kikuchi et al., 2002) were selected based on previously published studies. Cecal short-chain fatty acid contents were analyzed using HPLC [32].

### 2.7. Statistical Analysis

Data were analyzed via one-way ANOVA with Tukey’s Post Hoc test using SPSS v22.0 (IBM Corp., Armonk, NY, USA). Behavioral learning curves were assessed with two-way repeated-measures ANOVA. Sensory scores were analyzed with the Kruskal–Wallis test. *p* < 0.05 was considered statistically significant.

## 3. Results

### 3.1. Fermentation Performance and Probiotic Viability in Co-Culture

**Figure 1:**
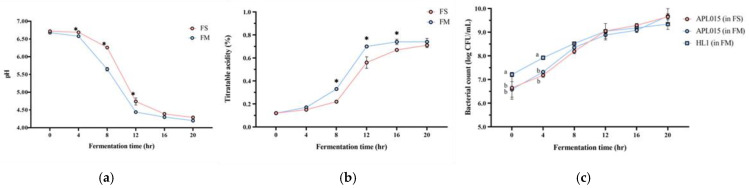
Changes in (**a**) pH value; (**b**) titratable acidity; and (**c**) viable bacterial count of different fermented milk products during fermentation. FS, *Lc. lactis* subsp. *cremoris* APL015 fermented milk; FM, mix of *Lb. kefiranofaciens* HL1 and *Lc. lactis* subsp. *cremoris* APL015 fermented milk. Asterisks (*) indicate significant differences between different products at each time point (*p* < 0.05), while different letters (a, b) indicate significant differences among samples within the same time point (*p* < 0.05). Data are shown as mean ± SD (n = 3).

Co-culturing *Lb. kefiranofaciens* HL1 with *Lc. lactis* APL015 effectively improved the milk fermentation behavior of HL1. HL1 alone (single-strain fermentation) exhibited minimal acidification, with pH dropping to only 5.3 ± 0.1 after 24 h; it failed to reach the desired endpoint of pH 4.6 even by 24 h. In contrast, APL015 alone rapidly fermented the milk, reaching pH 4.6 in 16.0 ± 0.5 h. The co-culture HL1 + APL015 (FM) also reached pH 4.6 in 16.3 ± 0.4 h (Figure 1a), essentially matching the acidification speed of APL015 alone. The final titratable acidity values were comparable between APL015-only and HL1 + APL015 fermented milk (0.78% vs. 0.80% lactic acid, *p* > 0.05) (Figure 1b). These results indicate that the presence of HL1 did not impede the acid-producing ability of APL015 in milk. Indeed, in laboratory broth co-culture tests, HL1 and APL015 appeared to stimulate each other’s metabolism, yielding lower pH than either alone. By eliminating the slow acidification issue of HL1, the co-culture strategy achieved a practical fermentation timeframe.

Viable cell counts of both strains in the finished products confirmed successful co-culture. HL1 alone grew poorly in milk, while APL015 alone achieved ~10^9^ CFU/mL by 16 h. In the FM co-culture, both strains were present at high levels: HL1 reached 10^8^ ± 0.2 CFU/mL and APL015 reached ~10^9^ ± 0.1 CFU/mL at the endpoint (Figure 1c, no significant difference between strains). Thus, HL1’s viable count in co-fermented milk was increased by over 100-fold compared to its monoculture in milk, demonstrating that APL015 effectively facilitated HL1 proliferation.

### 3.2. Physical and Sensory Properties of the Fermented Milk

**Figure 2:**
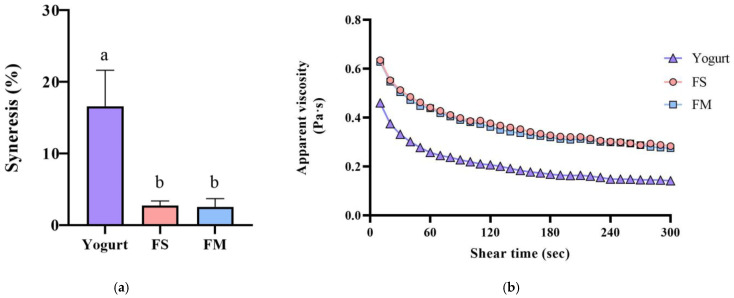
Syneresis (**a**) and apparent viscosity changes (**b**) of different fermented milk products. FS, *Lc. lactis* subsp. *cremoris* APL015 fermented milk; FM, mix of *Lb. kefiranofaciens* HL1 and *Lc. lactis* subsp. *cremoris* APL015 fermented milk. Different lowercase letters indicate significant differences among products (*p* < 0.05). Data are shown as mean ± SD (n = 3).

Co-culture of HL1 with APL015 produced a fermented milk with desirable rheological properties, similar to those fermented by APL015 alone. Both the FM (HL1 + APL015) and FS (APL015 only) samples formed a smooth, uniform coagulum with no visible whey separation after refrigeration. Syneresis tests quantified low whey separation for FM (≈23%) and FS (≈22%), both significantly lower than that of the reference yogurt made with standard yogurt culture (≈30% syneresis; *p* < 0.05) (Figure 2a). The viscosity of FM and FS at 4 °C (measured at 130 s^−1^) was high (approximately 400 mPa·s) and did not differ between the two; these were 1.5-fold higher than the viscosity of the reference yogurt (≈250 mPa·s) (Figure 2b). Texture profile analysis further showed that FM and FS had greater firmness and consistency compared to the reference yogurt (e.g., firmness 17.1 g in FM vs. 12.4 g in yogurt; *p* < 0.05) (Table 1), owing to the exopolysaccharide produced by APL015 (and possibly HL1) strengthening the gel structure. Crucially, no significant differences were detected between FM and FS in any texture parameter (firmness, cohesiveness, consistency, or syneresis; *p* > 0.05), indicating that inclusion of HL1 did not adversely affect the physical quality of the product. This is consistent with the earlier observation that HL1’s growth did not weaken curd formation by APL015.

**Figure 3:**
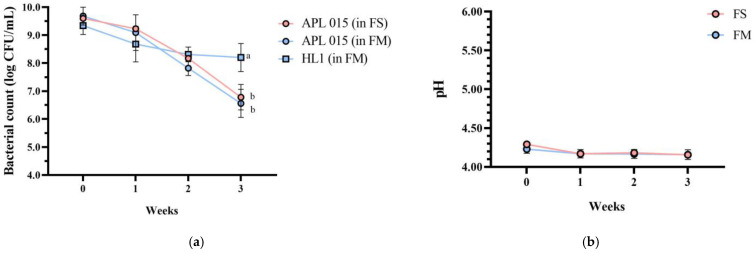

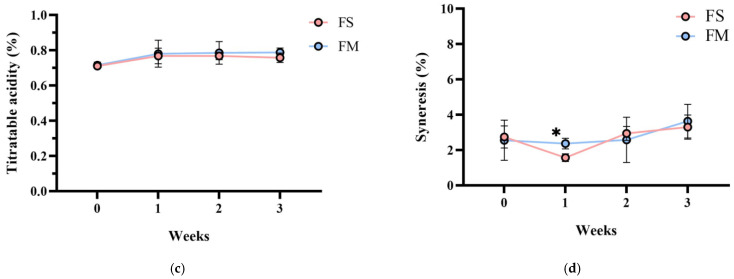
Changes in (**a**) bacterial count, (**b**) pH value, (**c**) TA, and (**d**) syneresis of different fermented milk products during storage stability test period for 3 weeks. FS, *Lc. lactis* subsp. *cremoris* APL015 fermented milk; FM, mix of *Lb. kefiranofaciens* HL1 and *Lc. lactis* subsp. *cremoris* APL015 fermented milk. Asterisks (*) indicate significant differences between different products at each time point (*p* < 0.05), while different letters (a, b) indicate significant differences among samples within the same time point (*p* < 0.05). Data are shown as mean ± SD (n = 3).

Importantly, product stability was good after 3 weeks of storage at 4 °C. No syneresis or texture breakdown was observed, and probiotic counts remained above 10^8^ CFU/mL (Figure 3). Overall, the co-culture product FM achieved the desired technological properties (rapid fermentation, thick consistency, appealing sensory profile) while successfully delivering a high dose of viable HL1.

### 3.3. Anti-Aging Effects in D-Galactose-Induced Aging Mice

#### 3.3.1. Behavioral Improvements in Learning and Memory

**Figure 4:**
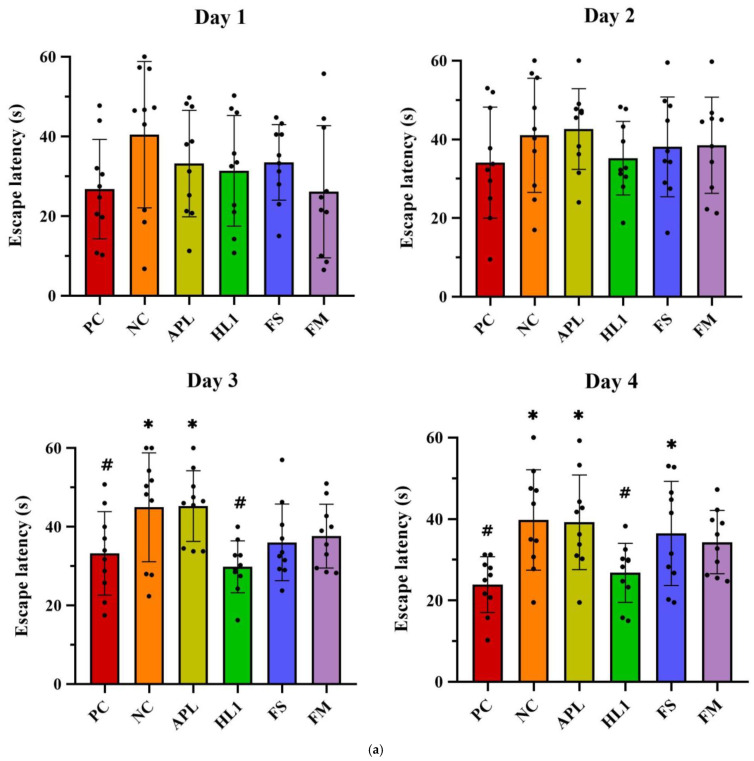

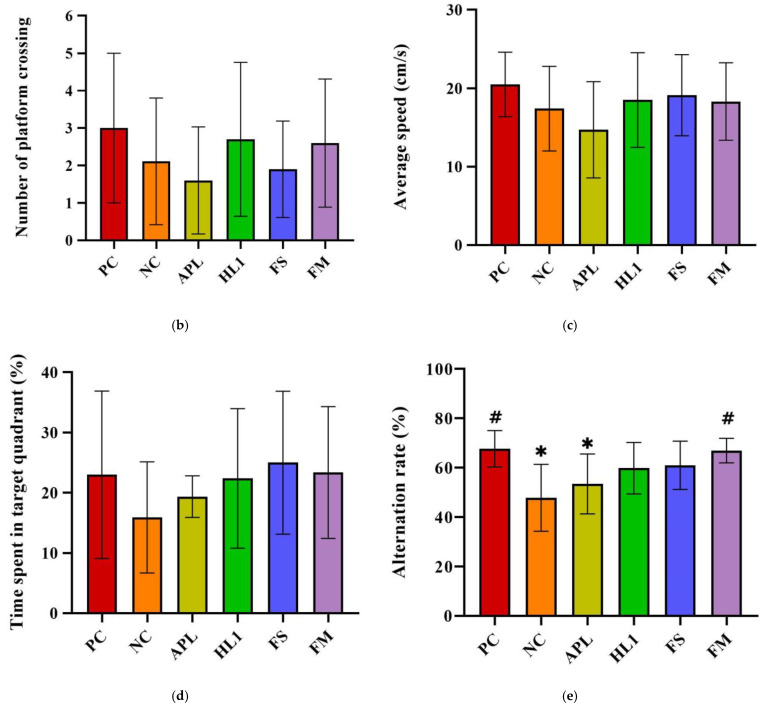
(**a**) Escape latency in Morris water maze test on different training days. The (**b**) number of platform crossings, (**c**) average speed, and (**d**) time spent in the target quadrant of mice in the Morris water maze probe test. (**e**) The alternation rate of mice in the Y-maze spontaneous alternation task. PC, positive control; NC, D-galactose-induced aging mice; APL, induced aging mice administered *Lc. lactis* subsp. *cremoris* APL015; HL1, induced aging mice administered *Lb. kefiranofaciens* HL1; FS, induced aging mice administered *Lc. lactis* subsp. *cremoris* APL015 fermented milk; FM, induced aging mice administered *Lb. kefiranofaciens* HL1 and *Lc. lactis* subsp. *cremoris* APL015 fermented milk. * indicates significant difference from PC (*p* < 0.05). # indicates significant difference from NC (*p* < 0.05). Data are shown as mean ± SD (n = 9–10 mice per group). Data were analyzed using the Kruskal–Wallis test followed by Dunn’s multiple comparisons test.

Chronic D-galactose administration induced marked behavioral impairments in the NC group, while mice treated with fermented milk (FM) or the probiotic strain HL1 showed significant cognitive improvements. In the Morris water maze test (Figure 4a), D-gal exposure severely disrupted spatial learning, as evidenced by longer swim paths and increased escape latencies during training in the NC group. In contrast, healthy control (PC) mice rapidly learned to locate the hidden platform. By day 4, mice in the HL1 group exhibited significantly shorter escape latencies than NC mice (*p* < 0.05), indicating enhanced spatial learning. The FM group also showed reduced latencies, comparable to HL1, although the difference between the two was not statistically significant. Mice in the APL015 (APL) and FS groups performed similarly to those in the NC group, with no significant improvement.

During the probe trial (platform removed), NC mice spent less time in the target quadrant (Figure 4b) and had fewer platform-site crossings (Figure 4c) than PC mice, indicating impaired memory retention. Both the HL1 and FM groups outperformed NC mice, spending significantly more time in the target quadrant and exhibiting more platform crossings (*p* < 0.05), suggesting improved long-term memory.

In the Y-maze spontaneous alternation test (Figure 4d), NC mice demonstrated a significantly reduced alternation rate (50.3 ± 5.4%) compared to PC mice (68.4 ± 6.1%; *p* < 0.001), confirming spatial working memory deficits. FM supplementation significantly improved alternation performance to 64.8 ± 5.0% (*p* < 0.01 vs. NC), nearly restoring it to control levels (no significant difference from PC). HL1 treatment alone led to a moderate but non-significant improvement (57.5 ± 6.8%; *p* = 0.09 vs. NC), while APL-treated mice remained similar to NC (52–53%, n.s.). These findings suggest that the FM product containing HL1 effectively ameliorated short-term memory impairments, with HL1 alone offering a modest, non-significant benefit.

#### 3.3.2. Oxidative Stress and Inflammatory Biomarkers

**Figure 5:**
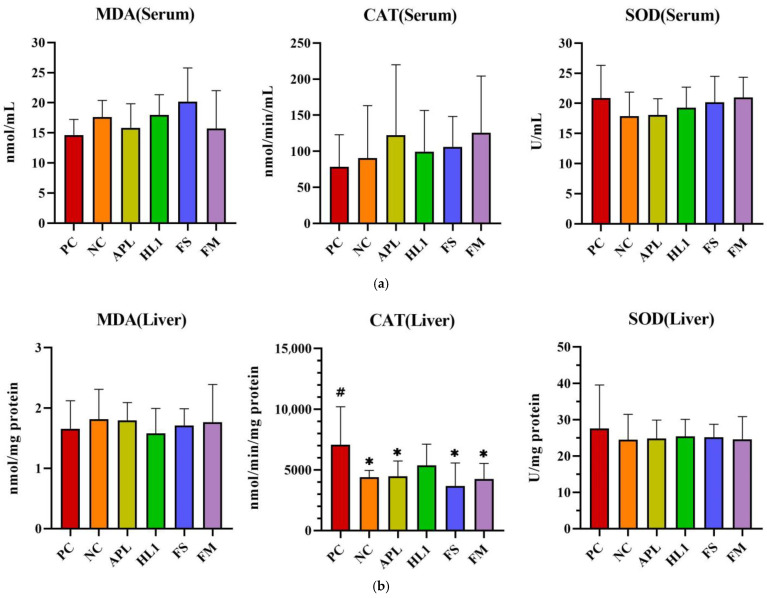

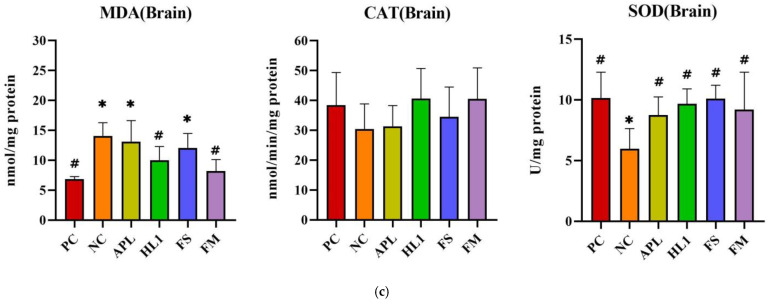
The activities of CAT and SOD and the levels of MDA in (**a**) serum, (**b**) liver, and (**c**) brain. PC, positive control; NC, D-galactose-induced aging mice; APL, induced aging mice administered *Lc. lactis* subsp. *cremoris* APL015; HL1, induced aging mice administered *Lb. kefiranofaciens* HL1; FS, induced aging mice administered *Lc. lactis* subsp. *cremoris* APL015 fermented milk; FM, induced aging mice administered *Lb. kefiranofaciens* HL1 and *Lc. lactis* subsp. *cremoris* APL015 fermented milk. * indicates significant difference from PC (*p* < 0.05). # indicates significant difference from NC (*p* < 0.05). Data are shown as mean ± SD (n = 9–10 mice per group). Data were analyzed Via the Kruskal–Wallis test followed by Dunn’s multiple comparisons test.

D-galactose administration induced pronounced oxidative stress and inflammation in NC mice, particularly within the central nervous system. This was evidenced by significantly elevated malondialdehyde (MDA) levels, a marker of lipid peroxidation, and reduced activity of superoxide dismutase (SOD), a critical anti-oxidant enzyme, in the brain compared to the healthy control group (PC) (Figure 5). Supplementation with *Lb. kefiranofaciens* HL1 or fermented milk (FM) effectively mitigated these alterations. Both interventions significantly increased SOD activity and reduced MDA concentrations in the brain relative to NC mice (*p* < 0.05), indicating enhanced anti-oxidant defense and reduced oxidative damage. In contrast, oxidative stress biomarkers in the serum and liver exhibited greater intergroup variability (Figure 5a,b), suggesting that D-galactose-induced redox imbalance was more severe in the brain than in peripheral tissues.

**Figure 6:**
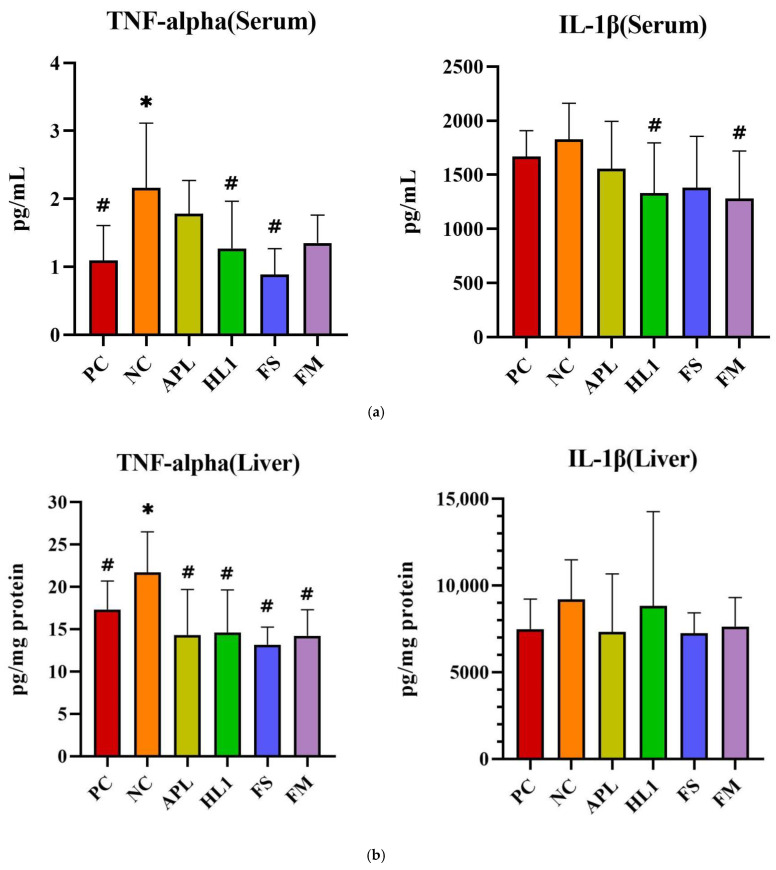

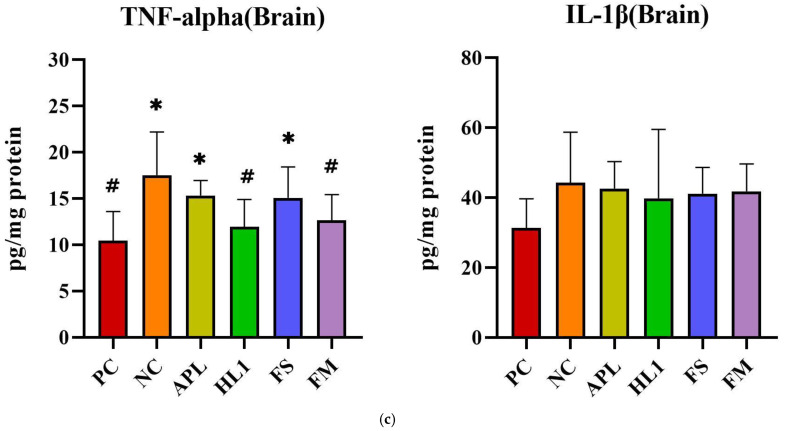
The levels of inflammatory cytokines in (**a**) serum, (**b**) liver, and (**c**) brain. PC, positive control; NC, D-galactose-induced aging mice; APL, induced aging mice administered *Lc. lactis* subsp. *cremoris* APL015; HL1, induced aging mice administered *Lb. kefiranofaciens* HL1; FS, induced aging mice administered *Lc. lactis* subsp. *cremoris* APL015 fermented milk; FM, induced aging mice administered *Lb. kefiranofaciens* HL1 and *Lc.* lactis subsp. *cremoris* APL015 fermented milk. * indicates significant difference from PC (*p* < 0.05). # indicates significant difference from NC (*p* < 0.05). Data are shown as mean ± SD (n = 9–10 mice per group). Data were analyzed via the Kruskal–Wallis test followed by Dunn’s multiple comparisons test.

The inflammatory response paralleled the oxidative stress pattern. NC mice displayed significantly elevated serum levels of pro-inflammatory cytokines TNF-α and IL-1β, indicative of systemic inflammation (Figure 6a). Treatment with HL1 or FM significantly reduced both cytokines (*p* < 0.05 vs. NC), restoring them toward levels observed in the PC group. In contrast, APL and FS failed to significantly reduce serum IL-1β. Hepatic TNF-α levels were also significantly increased in NC mice, and all treatment groups (APL, HL1, FS, and FM) achieved a significant reduction in this marker (*p* < 0.05 vs. NC), indicating that each intervention conferred some peripheral anti-inflammatory effect (Figure 6b). In the brain, neuroinflammation was most evident in the hippocampus, where D-galactose markedly increased TNF-α expression. Only the HL1 and FM treatments significantly attenuated this elevation (*p* < 0.05 vs. NC), demonstrating their neuroprotective potential (Figure 6c). Neither APL nor FS exerted a detectable effect on hippocampal cytokine levels, underscoring that the anti-inflammatory and neuroprotective actions were specific to interventions containing HL1.

Overall, mice that received the HL1-containing treatments (either as pure culture or in fermented milk) had markedly lower oxidative damage and inflammation than untreated D-gal mice, with levels often not significantly different from the healthy controls. The reductions in MDA and TNF-α and the increases in SOD activity suggest that HL1 helped bolster endogenous anti-oxidant systems and dampen pro-inflammatory signaling. These findings align with known anti-oxidant effects of HL1 observed in earlier studies and with reports on other probiotics mitigating oxidative injury associated with D-galactose. Notably, the fermented milk (FM) was at least as effective as HL1 alone in all these measures, indicating that delivering HL1 via co-fermentation did not diminish its bioactivity.

#### 3.3.3. Modulation of Gut Microbiota and SCFA Production

**Figure 7:**
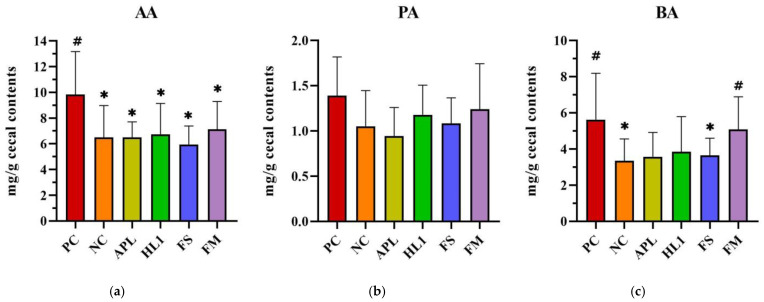
Analysis of short-chain fatty acids (SCFAs) in cecal contents: (**a**) acetic acid (AA), (**b**) propionic acid (PA), and (**c**) butyric acid (BA). PC, positive control; NC, D-galactose-induced aging mice; APL, aging mice administered *Lc. lactis* subsp. *cremoris* APL015; HL1, aging mice administered *Lb. kefiranofaciens* HL1; FS, aging mice administered APL015 fermented milk; FM, aging mice administered HL1 and APL015 co-fermented milk. Elevated SCFA levels, particularly butyrate, are associated with improved gut health and reduced inflammation. # indicates significant differences compared to NC (*p* < 0.05). and * indicates significant differences compared to PC (*p* < 0.05). Data are presented as mean ± SD (n = 8–10 mice per group). Statistical analysis was performed using the Kruskal–Wallis test followed by Dunn’s multiple comparisons test.

The analysis of specific taxa revealed that D-galactose treatment induced an overgrowth of opportunistic and potentially pathogenic bacteria (Figure 7). Notably, *Clostridium perfringens*, a toxin-producing anaerobe, was significantly reduced following supplementation with FM and FS compared to NC group. This reduction was not observed in the APL or HL1 groups, suggesting that the presence of metabolites after milk fermentation contributed to the observed effect. For Lactobacillus, HL1 treatment demonstrated a trend toward an increased abundance of this beneficial genus, although the difference was not statistically significant (*p* = 0.09).

**Figure 8:**
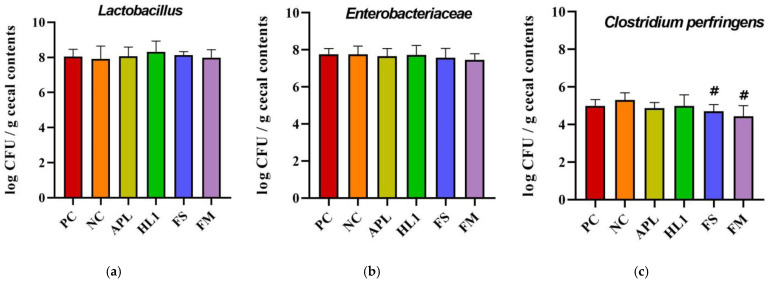
Analysis of cecal bacterial populations: (**a**) *Lactobacillus*, (**b**) *Enterobacteriaceae*, and (**c**) *Clostridium perfringens* counts (log CFU/g cecal contents). PC, positive control; NC, D-galactose-induced aging mice; APL, aging mice administered *Lc. lactis* APL015; HL1, aging mice administered *Lb. kefiranofaciens* HL1; FS, aging mice administered APL015 fermented milk; FM, aging mice administered HL1 + APL015 co-fermented milk. Suppression of *C. perfringens* and maintenance of *Lactobacillus* populations are indicators of improved gut microbiota composition and health status. Data are expressed as mean ± SD (n = 10 per group). Superscript symbol (#) indicate significant differences (*p* < 0.05) compared to NC. Data were analyzed via the Kruskal–Wallis test followed by Dunn’s multiple comparisons test.

Cecal SCFA analysis further corroborated these microbial findings (Figure 8). Mice in the NC group exhibited significantly lower butyric acid levels in cecal contents compared to the positive control (PC) group (~30% reduction), reflecting the negative impact of D-galactose on butyrate-producing bacteria. In FM-treated mice, butyrate concentrations were elevated to 5.0 ± 1.1 mg/g, effectively restoring butyrate levels to those observed in the NC group. There were no significant differences in acetate and propionate levels between FM-treated mice and NC controls. The HL1-alone group demonstrated a trend of increased butyrate levels compared to the NC group, though this difference did not reach statistical significance (*p* = 0.12). In contrast, the FS group (fermented milk without HL1) exhibited similar butyrate levels to the NC group. These findings suggest a synergistic effect of HL1 in the context of the co-cultured fermented milk matrix, with HL1 being particularly effective in promoting a gut environment enriched in butyrate.

In summary, the fermented milk containing HL1 brought about favorable modifications in gut microbiota composition: it suppressed potentially harmful bacteria (such as *C. perfringens*) and boosted populations that contribute to gut homeostasis and SCFA production. These changes correlated with the higher levels of butyrate found in FM mice. Butyrate is a key metabolite known to strengthen the intestinal barrier and exert systemic anti-inflammatory effects, as well as influence brain function via the gut–brain axis. The gut microbial shifts were less pronounced in mice given HL1 alone, suggesting that the fermented milk matrix and co-culture context enhanced HL1’s impact on the microbiome.

## 4. Discussion

In this study, we successfully developed a novel probiotic fermented milk incorporating *Lb. kefiranofaciens* HL1 by co-culturing it with *Lc. lactis* subsp. *cremoris* APL015 and demonstrated that this product confers significant anti-aging and anti-oxidative benefits in an accelerated aging mouse model. The co-culture strategy effectively addressed the technological challenge of using HL1 in dairy fermentation. *Lb. kefiranofaciens* alone could not acidify milk adequately, echoing previous reports of its poor performance in milk culture [20]. By pairing HL1 with the vigorous acid-producer APL015, we achieved normal fermentation kinetics and curd formation without compromising the viability of HL1. Indeed, APL015 proved to be an optimal partner that not only allowed HL1 to survive but also possibly stimulated its metabolism. APL015 is an exopolysaccharide-producing *Lactococcus*, which likely contributed to the improved texture (low syneresis and high viscosity) of the fermented milk. The ropy polysaccharides from *Lc. lactis* subsp. *cremoris* is known to enhance water-holding in the gel and impart a creamy mouthfeel [33]. This was evident in our texture analysis, where both FS and FM (with APL015) outperformed a standard yogurt in physical stability. Importantly, the inclusion of HL1 did not negatively impact these qualities. The FM product was texturally indistinguishable from the APL015-only counterpart, indicating that HL1’s EPS (kefiran) [34] and APL015’s EPS likely complemented each other in building the gel matrix.

The in vivo results provide compelling evidence that the fermented milk exerts anti-aging effects, and they highlight the multifaceted role of the gut–brain axis. D-galactose treatment is known to induce a state of accelerated aging characterized by memory impairment, increased oxidative damage, and elevated pro-inflammatory cytokines. In our study, mice that received the HL1-containing fermented milk were protected against many of these changes. The FM group’s performance in the Y-maze (working memory) was on par with healthy controls, suggesting near-complete prevention of the hippocampal dysfunction induced by D-gal. The HL1-alone group, while also beneficial, had a smaller effect on Y-maze alternation, which might indicate that continuous presence of HL1 in the gut (via daily feeding of fermented milk) or the matrix of the fermented milk provided added benefit for short-term memory. Conversely, HL1 alone significantly improved water maze learning speed, an effect consistent with prior findings that *Lb. kefiranofaciens* HL1 enhances cognitive function. Ho et al. [11] reported that a probiotic mixture containing *Lb. kefiranofaciens* improved learning and memory in D-galactose-treated mice. Our results corroborate those findings, now using HL1 as a single (or primary) probiotic in a food-based delivery form. The difference in which test (Y-maze vs. water maze) showed stronger improvement with HL1 vs. FM could be due to variations in how HL1’s metabolites affect different memory processes, or simply the timing of effects. It is possible that the co-fermented milk, which alters gut microbiota more substantially, preferentially affects short-term memory via gut–brain signaling (e.g., vagal pathways or immune modulation), whereas high doses of HL1 alone might directly impact brain function via absorbed metabolites. Regardless, both approaches improved cognition, reinforcing the potential of HL1 as a neuroprotective probiotic.

A central finding is that the fermented milk attenuated oxidative stress and inflammation, key drivers of aging. HL1 has demonstrated anti-oxidative activity in vitro, but here we show it can also boost anti-oxidant defenses in vivo. Brain SOD activity was restored in HL1- and FM-treated mice, suggesting activation of endogenous anti-oxidant enzymes. This could be mediated by the Nrf2 pathway, a master regulator of cellular anti-oxidant response [35]. Our results (higher SOD, lower MDA) align with those on Nrf2-mediated effects and with other probiotic studies where Nrf2 signaling was upregulated [36,37]. It is plausible that HL1 (especially in fermented milk) increased levels of certain microbial metabolites such as butyrate, which is known to activate Nrf2 and other cytoprotective pathways.

Concurrently, the reduction in pro-inflammatory cytokines (IL-1β, TNF-α) in the serum, liver, and brain indicates an anti-inflammatory effect. Chronic low-grade inflammation was significantly blunted by HL1 and FM. Lower brain TNF-α suggests protection against D-gal-induced neuroinflammation. This likely contributed to the preservation of cognitive function, as neuroinflammatory cytokines can impair synaptic function and memory. It is worth noting that the APL015-only groups did not show these biochemical improvements, which underscores that the anti-aging effects are attributable to the functional properties of HL1 in particular rather than just any fermented milk or general nutritional effect. APL015 is not known to have specific anti-oxidant effects, and our results confirm that it provided minimal protection by itself. Thus, the probiotic HL1 is the key bioactive agent, and the co-culture product is essentially a delivery vehicle that ensures the viability of HL1 and potentially adds complementary benefits.

One of the most intriguing aspects of this work is the observed modulation of the gut microbiota. D-galactose has been reported to cause gut dysbiosis and compromise the gut barrier, possibly allowing endotoxins to exacerbate inflammation [38]. We found that feeding mice the HL1 fermented milk shifted the microbiota towards a healthier profile: harmful bacteria such as *C. perfringens* were suppressed, and beneficial SCFA producers grew (as evidenced by increased cecal butyrate). *C. perfringens* is a toxin-producing bacterium that can damage the gut lining and trigger inflammation [39]. Its reduction in FM mice could mean less translocation of toxins and reduced inflammatory stimulation. Additionally, butyrate is renowned for its anti-inflammatory and neuroprotective roles [40]. It strengthens the gut barrier [41], reduces peripheral inflammation [42], and even enters circulation to influence microglia and neurons [43]. The elevated butyrate in FM mice likely acted on multiple fronts, including locally to improve gut integrity and systemically to activate anti-oxidative pathways and modulate brain function. In fact, a growing evidence base links increased SCFA levels to improved cognition in aging models [44,45]. Our data suggest the HL1 fermented milk created a gut environment conducive to SCFA production, an advantage that HL1 alone did not fully replicate. This could be due to the presence of APL015 and fermentation-derived substrates (e.g., lactate and EPS) that feed butyrogenic commensals. EPS from *Lactococcus* and *Lactobacillus* can function as a prebiotic, stimulating the growth of *Butyrivibrio* and other fiber-utilizing microbes [46,47]. Therefore, the combination of HL1 and APL015 in a fermented matrix may have synergized to reshape the microbiome in a way that favors an anti-aging milieu.

The improvements in gut microbiota and metabolite profiles in turn likely fed back to reduce systemic inflammation and neuroinflammation, forming a virtuous cycle of gut–brain axis modulation. It is noteworthy that improvements in behavior correlated with these biochemical changes: The FM group, which had the greatest microbiota improvements (high butyrate, low *C. perfringens*), also showed the best working memory performance. Meanwhile, the HL1 group, with somewhat fewer microbiota changes, still had substantial anti-oxidant and anti-inflammatory effects and showed better reference memory (water maze). Together, these outcomes underscore that the mechanism of action for the fermented milk involves both direct probiotic effects (anti-oxidant enzyme induction) and indirect effects via microbiota modulation and metabolite production. This is consistent with the concept of postbiotics and metabolite-mediated probiotic benefits. HL1 might produce or stimulate the production of SCFAs, in addition to other metabolites such as polyamines or peptides that combat oxidative stress. Additionally, kefiran (the EPS from HL1) itself has been reported to have anti-oxidative and immunomodulatory properties [48]. APL015’s EPS could similarly contribute by preventing pathogen adhesion or by acting as fermentable fiber for beneficial microbes.

From a broader perspective, our findings highlight the potential of using fermented functional foods to combat age-related cognitive decline and inflammation. Compared to administering isolated probiotic capsules, fermented milk offers a food-based strategy that might have higher acceptability and additional nutrients. The fermentation process can generate bioactive peptides [49] that might synergize with probiotic action. While we did not specifically measure such peptides, it is possible that they played a minor role in the benefits of FS/FM. However, since FS (APL-only fermented milk) did not significantly improve the aging biomarkers, it appears that the presence of the probiotic HL1 was the critical factor for efficacy.

This study is among the first to demonstrate the anti-aging effects of a kefir-derived probiotic delivered in a dairy product. Previous studies with kefir or its isolates have shown health benefits such as immunomodulation and anti-fatigue effects, and particularly *Lb. kefiranofaciens* M1 has known anti-inflammatory [50,51] and anti-allergic [52,53] effects in mice. Our work extends these insights to aging, showing that *Lb. kefiranofaciens* HL1 can counteract aging-related oxidative brain damage and memory decline. The use of a co-culture product addresses the practicality issue. It demonstrates a way to incorporate such strains into functional foods. Mechanistically, future studies could delve deeper into how HL1 influences the host at the molecular level. Measuring brain expression of neurotrophic factors or signaling pathways (Nrf2, NF-κB) would shed light on gut–brain communications. Our results strongly suggest an involvement of Nrf2 and reduced NF-κB activity.

In conclusion, the co-fermented milk containing *Lb. kefiranofaciens* HL1 and *Lc. lactis* APL015 emerged as a multifunctional functional food—it not only delivers live probiotics at efficacious levels but also improves gut health parameters and exerts systemic anti-oxidant and anti-inflammatory effects, all of which translated into better cognitive performance in aged mice. This aligns with the growing evidence that certain probiotics can ameliorate age-related oxidative damage and cognitive decline. In this study, the term “anti-aging” specifically refers to improvements in oxidative stress, inflammation, and cognitive function. Our study adds a novel candidate to that list (HL1) and provides a practical means to consume it. While this animal study provides promising results, translation to human applications warrants further investigation. Human equivalent dosages should be carefully calculated, considering differences in metabolism and microbiota between species. Additionally, food formulation for human use must address sensory acceptability, regulatory compliance regarding health claims, and probiotic viability during storage. It should also be noted that the observed effects are strain-specific and may not be generalized to other *Lb. kefiranofaciens* or *Lc. lactis* strains without similar properties. Furthermore, this study did not include human sensory evaluation, which would be necessary for future product development.

## 5. Conclusions

In summary, the fermented milk containing HL1 and APL015 demonstrated potent anti-aging and anti-oxidative properties in vivo, highlighting the synergistic value of this probiotic co-culture. Our findings underscore the importance of the gut–brain axis in aging and suggest that functional fermented foods targeting gut health can impart cognitive and systemic benefits. This work paves the way for the further development of HL1-based functional foods or nutraceuticals aimed at promoting healthy aging. Future studies should explore the efficacy of this fermented milk in human trials and investigate the molecular mechanisms (such as Nrf2 activation and gut–brain signaling) underlying its protective effects. The novel HL1 fermented milk represents a promising, natural dietary strategy to combat oxidative stress and inflammation associated with aging, potentially improving quality of life for the elderly.

## Figures and Tables

**Table 1:** Texture attributes of different fermented milk products.

Fermented Milk Type	Firmness (g)	Cohesiveness (g)	Consistency (g·s)	Viscosity Index (g·s)
Yogurt	145.36 ± 19.29 ^b^	57.53 ± 4.05	2438.20 ± 241.34 ^b^	826.37 ± 72.26
FS	171.95 ± 17.05 ^a^	56.40 ± 5.82	2770.47 ± 219.75 ^a^	815.89 ± 49.63
FM	171.32 ± 10.65 ^a^	58.82 ± 4.45	2838.27 ± 179.20 ^a^	855.90 ± 31.03

FS, fermented milk with *Lactococcus lactis* subsp. *cremoris* APL015; FM, co-fermented milk with *Lactobacillus kefiranofaciens* HL1 and *Lc. lactis* subsp. *cremoris* APL015; Yogurt, fermented milk with *Lactobacillus bulgaricus* and *Streptococcus thermophilus.* ^a, b^ Values with different superscript letters in the same row are significantly different (*p* < 0.05). Data are expressed as mean ± SD (n = 3). Statistical analysis was performed using one-way ANOVA followed by Tukey’s multiple comparison test.

## Data Availability

All data supporting the findings of this study are contained within the article; further inquiries can be directed to the corresponding author.

## Abbreviations

APL15	*Lactococcus lactis* subsp. *cremoris* APL015
FM	co-cultured fermented milk
SCFA	short-chain fatty acid
